# Root-Applied Cerium Oxide Nanoparticles and Their Specific Effects on Plants: A Review

**DOI:** 10.3390/ijms25074018

**Published:** 2024-04-04

**Authors:** Monika Pietrzak, Elżbieta Skiba, Wojciech M. Wolf

**Affiliations:** Institute of General and Ecological Chemistry, Lodz University of Technology, Zeromskiego 114, 90-543 Lodz, Poland; wojciech.wolf@p.lodz.pl

**Keywords:** plants, nanoceria, physiological activity, metabolism, oxidative stress, photosynthesis

## Abstract

With the pronounced increase in nanotechnology, it is likely that biological systems will be exposed to excess nanoparticles (NPs). Cerium oxide nanoparticles (CeO_2_ NPs) are among the most abundantly produced nanomaterials in the world. Their widespread use raises fundamental questions related to the accumulation in the environment and further interactions with living organisms, especially plants. NPs present in either soil or soilless environments are absorbed by the plant root systems and further transported to the aboveground parts. After entering the cytoplasm, NPs interact with chloroplast, nucleus, and other structures responsible for metabolic processes at the cellular level. In recent years, several studies have shown the impact of nanoceria on plant growth and metabolic processes. Research performed on different plants has shown a dual role for CeO_2_ NPs. The observed effects can be positive or negative and strongly depend on the plant species, characterization, and concentrations of NPs. This review describes the impact of root-applied CeO_2_ NPs on plant growth, photosynthesis, metal homeostasis, and parameters of induced oxidative stress.

## 1. Introduction

The rapid expansion of nanotechnology has led to many applications of engineered nanoparticles (ENPs) in technology, medicine, and agriculture [1]. This widespread use has resulted in nanoparticles becoming increasingly abundant in the environment. The steadily growing production and logistics make either controlled or unintended releases to soil, water, and air environments very likely. Therefore, ENPs have become essential environmental stress factors of growing importance [2].

Cerium oxide nanoparticles (CeO_2_ NPs) are often used as additives to commercial products, like fuel additives, cosmetics, and electronic devices [3,4]. According to the highly appreciated Allied Market Research, the global market for CeO_2_ NPs was valued at USD 385.2 million in 2020 [5]. Therefore, the increased production and consumption of nanoceria lead to its migration to the terrestrial environment [6]. The natural level of Ce in surface soils has been estimated, on average, at 56.7 mg/kg [7]. However, much higher levels have also been detected [8,9].

Plants are essential components of terrestrial ecosystems and are highly exposed to a plethora of stressing factors. As far as food production is concerned, the primary absorbers for nanoparticles are arable land areas [10]. Several mechanisms are followed by nanoparticles to enter the plant cell interior. Regardless of the limited cell wall’s pore sizes (5–20 nm), there is firm evidence that larger nanoparticles are able to overcome that barrier [11]. Unfortunately, the precise mechanism of NPs absorption by roots and their further transport has not yet been fully established, with some ambiguous results published to date [12]. Notably, the Casparian strip serves as a barrier to the movement of extracellular ions in plant roots, limiting their transport to the xylem via apoplast [13]. Its role in regulating the movement of cerium oxide nanoparticles is complex and depends on diverse factors, including nanoparticle size, surface chemistry, root anatomy, and the physiological effects on plants. The available experimental data indicate that—upon the root uptake—NPs concentrations in plants have the following order: roots > shoots > fruits > seeds [14].

Several possible mechanisms for the toxic effects of NPs in biological systems have been described in the scientific literature so far. They often involve the direct “binding” of nanoparticles on the surface of cells, allowing them to interact with lipids, proteins, and other components of biological membranes [15]. The toxicity of nanoparticles may also result from the dissolution process and the following release of toxic metal ions [16]. This is relevant at either the environmental or cellular level and, finally, it may prompt the generation of reactive oxygen species (ROS) in the plant organs. Overproduction of these highly reactive entities may surpass the efficiency of the plant's cellular antioxidant systems. In particular, the excessive production of ROS can cause lipid peroxidation or oxidation of nitrogenous bases and deoxyribose in the DNA structure [17]. Following the structural and physicochemical properties of NPs, Metcalfe et al. [18] characterized four potential mechanisms of toxic effects induced by NPs. The toxicity of NPs may be directly related to their size and shape. The particle size, on the order of 10^−9^ m, makes it easier to cross the barrier of biological membranes and penetrate the organism, while the shape of NPs can be a steric factor that blocks the active centers of proteins, phospholipids, and nucleic acids.

The surface properties of NPs are often responsible for their toxic effects. Specifically, the high reactivity at the surface, their photochemical properties, and charge density are important factors that affect metabolic processes involved by ROS at the cell level. Metallic nanoparticles can release ions from their surface and be carriers that facilitate toxic substances to enter plant organisms [19,20,21,22]. Notably, there is no common mechanism that can be applied to explain the toxicity of a plethora of either anthropogenic or geogenic NPs existing in terrestrial environments [16,23,24].

**Figure 1:**
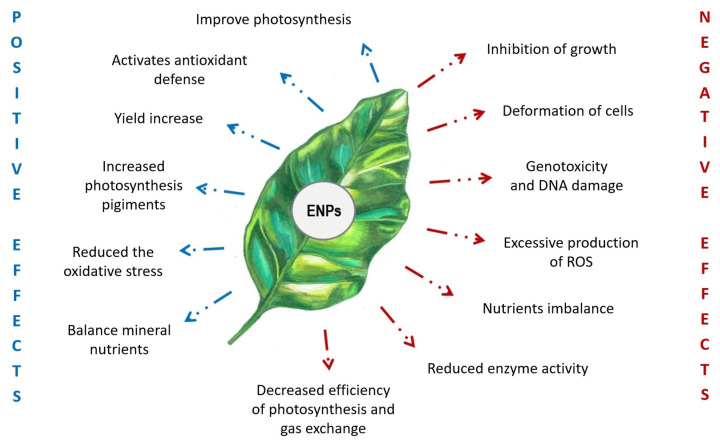
Plant responses to cerium oxide nanoparticles. Positive and negative effects are in blue and red, respectively.

It is well known that cerium oxide nanoparticles have an ambient effect on plants (Figure 1). In particular, cultivation experiments with plants subjected to foliar or root treatments with nanoceria showed that the accumulation of NPs in plant tissues directly affected plant growth, photosynthesis, and mineral nutrition. The equilibrium between reactive oxygen species and the plant antioxidant system was also affected [11].

Details of investigations on plants subjected to nanoceria exposures are summarized in Table 1. 

The species nomenclature validation was adopted from the World Flora Online (WFO) [56]. This review covers experiments on the root exposure of CeO_2_ NPs to plants. Our work focuses on papers published from 2012 onward. The following keywords were used in the search “cerium oxide nanoparticles plants”, “nanoceria plants”, “cerium oxide nanoparticles photosynthesis”, and “cerium oxide nanoparticles plant metabolism”. We investigated major databases, i.e., ScienceDirect, Scopus, SciVal (Elsevier, Amsterdam, The Netherlands), SciFinder (American Chemical Society, Columbus, The United States of America), Springer (Springer Nature, Berlin, Germany), Science (American Association for the Advancement of Science, Washington, The United States of America). Papers related to the foliar applications of CeO_2_ NPs were excluded. The distribution of nanoceria levels in soil cultivation and hydroponic media, as reviewed in this work, is presented in the Supplementary Materials (Figure S1). In particular, Kulak [57] emphasized that the potential effects of CeO_2_ NPs are concentration-dependent and levels above 100 mg/L could be phytotoxic. We suggest accepting this value as a low limit for CeO_2_ NPs presence in the growing environment.

## 2. The Influence of Cerium Oxide Nanoparticles on the Process of Plant Growth and Development-Literature Review

### 2.1. Plant Growth and Biomass Production

Wang et al. [47] studied the effect of nanometric cerium oxide (0.1, 1 and 10 mg/L) on the growth parameters of hydroponically grown tomatoes (*Solanum lycopersicum* L.). The experiment spanned 70 days and involved cultivating plants treated with CeO_2_ NPs. The study included seed germination, vegetative growth, and generative growth. The authors proved that CeO_2_ in the tested concentration range did not affect either the rate of seed germination or the development of leaves and flowers. The yields of the fresh and dry biomass were also not affected. On the other hand, at the highest concentration of CeO_2_ NPs (10 mg/L), a significantly faster plant growth rate was observed, as well as an increased yield of tomatoes. The inductively coupled plasma mass spectrometry (ICP-MS) analysis showed that the accumulation of Ce in plant organs correlated with the increased concentration of CeO_2_ NPs in the growing media. Roots showed the highest Ce accumulation capacity (even 12 µg/g Ce), followed by tomato stems, leaves, and fruits. The ability to store Ce in edible parts of plants was also confirmed by Zhang et al. [51], who treated radish ((*Raphanus raphanistrum* subsp. *sativus* (L.) Domin) with nanometric CeO_2_ at a concentration of 10 mg/L. However, the stressor did not affect the plant biomass. No visible changes in the morphological structure of plants under nanoceria administration were observed. No significant changes in biomass production were noticed in sunflower (*Helianthus annuus* L.) [46], basil (*Ocimum basilicum* L.) [28], and canola (*Brassica napus* L.) [29] cultivated in soils and exposed to CeO_2_ NPs, when the concentrations of nanoparticles added to the growth media were 100–800 mg/kg, 80 mg/kg and 500 mg/kg, respectively. Hernandez-Viezcas et al. [39] found no symptoms of CeO_2_ NPs toxicity in mesquite (*Neltuma velutina (Wooton) Britton & Rose*), which is a plant typical to dry habitats. Despite relatively high concentrations of nanoparticles (500, 1000, 2000 and 4000 mg/L), no visible symptoms of toxicity, such as chlorosis or growth inhibition, were observed in hydroponically cultivated plants.

Numerous studies have proved that the influence of CeO_2_ NPs on plant growth and biomass production is closely related to the concentration of nanoparticles in the soil or the nutrient solution and is dependent on the type of crop. It is commonly known that low concentrations of rare earth elements (REE), including cerium, have a positive impact on plant growth and cultivation yield, but have resulted in toxicity at higher concentrations [58,59]. This effect was reported by Gui et al. [36] for nanometric CeO_2_. It significantly increased the biomass of lettuce (*Lactuca sativa* L.) after 30 days of soil supplementation at 100 mg/kg CeO_2_ NPs. However, a lower dose of nanoceria (50 mg/kg) did not affect the growth of plants, while a concentration of 1000 mg/kg radically reduced the dry weight of roots and shoots. Additionally, the promotion of plant growth at low concentrations of CeO_2_ NPs was also detected in hydroponic pot experiments. According to Abbas et al. [49], CeO_2_ nanoparticles at concentrations of 100 and 500 mg/L stimulated the production of fresh biomass of either the roots or shoots of wheat (*Triticum aestivum* L.), while higher doses (1000 and 2000 mg/L) decreased plant biomass. As indicated by the authors of the study, the beneficial effect of CeO_2_ NPs at a relatively low concentration in the plant growth medium may be related to the enhanced efficiency of the photosynthesis process resulting in a higher content of macronutrients in plant tissues, which ultimately translates into biomass production. In thale cress (*Arabidopsis thaliana* (L.) Heynh.), cultivated in agar culture [55], the toxic effect of CeO_2_ NPs was observed at concentrations higher than 1000 mg/L, while the lower concentrations (< 500 mg/L) stimulated the production of root and shoot biomass. The authors emphasized that the inhibition of biomass production (observed for the highest doses of NPs) was not due to Ce ions released from CeO_2_ NPs in the growth medium, but to its nanometric form. The impact of different forms of cerium (cerium oxide nanoparticles, bulk cerium oxide, and ionic cerium nitrate) on plant development, as well as the uptake and further translocation of Cu, Mn, Zn, and Fe by sugar pea (*Lathyrus oleraceus* Lam.), was investigated by Skiba and Wolf [60]. The authors proved that at a cerium level of 200 mg/L, the strongest impact on plant growth and the metal uptake (Cu, Mn, Zn, Fe) was observed for supplementation with ionic cerium nitrate; on the contrary, the weakest was observed in the bulk cerium form. The phenomenon where a given factor (e.g., a toxin) has contradictory effects on the body, depending on the dose used (e.g., low concentrations of a given substance cause positive physiological reactions in the organism, while high doses generate toxic effects), is defined in scientific literature as hormesis [61]. The effect of the stressor dose on the growth and development of plants depends, in particular, on the species used in the experiment. Rossi et al. [30] noted an increase in the root biomass of canola (*Brassica napus* L.) cultivated in contact with 1000 mg/kg CeO_2_ NPs, while it was not significantly affected by a lower dose of nanoparticles (200 mg/kg). The biomass of green parts was comparable to the controls. Regarding cilantro (*Coriandrum sativum* L.) cultivated in soil augmented with nanoceria (62.5, 125, 250 and 500 mg/kg), a positive effect of CeO_2_ NPs on the length of roots and shoots was observed only at the concentration of nanoparticles in the soil equal to 125 mg/kg. Although plant growth was boosted, no significant changes in biomass production were observed. Other applied doses of CeO_2_ NPs did not change the plant growth parameters, presenting results close to the reference values [31].A number of studies discuss the effect of CeO_2_ NPs on the yield of crops, where the treatment time with nanoparticles includes the generative phase of plant growth. In the experiment carried out by Rico et al. [27], barley (*Hordeum vulgare* L.) was grown for 169 days in soil supplemented with CeO_2_ nanoparticles at low (125 mg/kg), medium (250 mg/kg), and high (500 mg/kg) concentrations. The results of the experiment showed that the lowest tested concentration of nanoceria did not significantly affect barley growth parameters, keeping them at a level close to the control. The medium CeO_2_ NPs dose reduced the number of spikes, but the quantity and weight of grain increased. At a concentration of 500 mg/kg, it was observed that the plants did not develop fully mature spikes, although the height, dry weight, and water content of the plants were much higher in comparison to control plants. The effect of CeO_2_ NPs on barley yield was also studied in the experiment conducted by Marchiol et al. [26]. After 92 days of cultivation with nanoparticles, the authors observed the nanoceria effect on reducing the leaf size and the number of spikes per plant. Moreover, CeO_2_ NPs significantly extended the vegetative phase of plant growth. Rico et al. [48] observed that CeO_2_ NPs in the tested concentration range (125, 250 and 500 mg/kg) prolonged the spike formation phase and the achievement of physiological maturity in wheat (*Triticum aestivum* L.). The presence of nanoparticles in the soil had a positive impact on the yield parameters such as the number of seeds per spike or grain weight. The observed effect depends on nanoceria concentration in the soil. The highest values of yield parameters were observed with 500 mg/kg CeO_2_ NPs supplementation. Furthermore, Du et al. [50] observed that CeO_2_ NPs extended the flowering phase of wheat and shortened the period of grain formation. Despite these changes, no impact of nanoceria on biomass production or plant yield parameters was noted. Zhao et al. [38] conducted an experiment involving the cultivation of maize (*Zea mays* L.) to full maturity in soil amended with CeO_2_ NPs. In the studied NPs concentrations (400 and 800 mg/kg), no signs of toxicity were found in plants, manifested by the interference of CeO_2_ NPs in biomass production, plant height, leaf size, or cob development. CeO_2_ nanoparticles at the same concentration range did not induce differences in the growth parameters of cucumber (*Cucumis sativus*) [32]. Dry biomass, the length of shoots, and the leaf area of plants treated with CeO_2_ NPs were similar to those found in the control plants. Significant changes were observed in the cucumber fruit weight, which was lower by 32% at 800 mg/kg CeO_2_ NPs compared to the reference value.

Results from many experiments demonstrate the ability of CeO_2_ NPs to mitigate environmental stress impacts in plants and ultimately affect the obtained plant biomass. Rossi et al. [29] proved that nanoceria can alleviate the negative effects of salinity stress in canola (*Brassica napus* L.) by modifying the cell wall of the roots, thus facilitating the transport of Na^+^ from canola roots to shoots. Nanoparticles can also diminish the heavy metal stress in plants by (1) reducing the bioavailability of these metals in the soil, (2) regulating the expression of genes responsible for their transport, (3) strengthening antioxidant systems, or (4) stimulating the secretion of organic acids or metal chelators into the soil [62]. According to Rossi et al. [63], nano-sized cerium (IV) oxide prevents Cd translocation into soybean (*Glycine max* (L.) Merr.) shoots, probably by chelation of Cd and sequestration in the root cell vacuole. Wang et al. [64] reported that CeO_2_ NPs reduce the inhibition of chlorophyll biosynthesis, resulting from the presence of CdCl_2_ in the hydroponic solution. Additionally, nanoceria minimizes the negative effects of NaCl, related to the reduction of fresh biomass in rice (*Oryza sativa* L.) seedlings. These results could be due to the antioxidative properties of CeO_2_ NPs, which support plant defense mechanisms, and to the overproduction of ROS induced by CdCl_2_ and NaCl stress.

### 2.2. Mineral Nutrition

The uptake of nutrients by plants is determined by the availability of elements, the ability of plants to accumulate them, and the interactions between them (antagonism, synergism). The literature indicates that plant growth in a medium supplemented with nanoparticles can significantly affect metal homeostasis and modify the nutritional value of plants [65].

Corral-Diaz et al. [52] evaluated mineral nutrition in radish ((*Raphanus raphanistrum* subsp. *sativus* (L.) Domin) at both initial and final growth stages (12 and 40 days after planting, respectively). In the first period, CeO_2_ nanoparticles affected the uptake of minerals by altering B, Mo, P, S, Cu, and Zn accumulations compared to the control. In plants grown to full maturity, no effect of CeO_2_ NPs on the level of accumulation of micro- and macro elements in the leaves or edible storage root of radish was found. However, the nanoparticles significantly reduced the accumulation of S in the roots, which was visible at all tested doses of Ce (62.5, 125, 250 and 500 mg/kg). As suggested by the authors, this may be due to the blocking of the uptake of sulfur in the form of SO_4_^2-^ ions, caused by the formation of Ce(SO_4_)_2_ in the soil solution. Rossi et al. [30] compared the levels of selected macronutrients in the roots and leaves of canola (*Brassica napus* L.) cultivated as a control to those treated with nanometric CeO_2_. In the examined NPs concentrations (200 and 1000 mg/kg), the nanoparticle addition led to increased Mg accumulation in canola leaves and prompted higher chlorophyll levels. The effect of nanometric cerium oxide on the levels of selected macro elements in roots and shoots of wheat (*Triticum aestivum* L.) was investigated by Abbas et al. [49], based on soilless plant cultivation. In the studied concentration range (100, 500, 1000 and 2000 mg/L), CeO_2_ NPs reduced nitrogen uptake and further accumulation in the green parts of plants. At the same time, the nanoparticles promoted potassium utilization by increasing its levels in roots and shoots. The effect of CeO_2_ NPs on the accumulation of phosphorus depended on the NPs concentration in the nutrient solution. Compared to the reference, statistically significant changes in the level of phosphorous in roots were observed at 500 mg/L CeO_2_ NPs. At the highest tested concentration (2000 mg/L), phosphorus content in the roots and shoots decreased by 10% and 19%, respectively, compared to the control plants. The influence of nanometric CeO_2_ on metal homeostasis in plants was also assessed by Peralta-Videa et al. [44]. The experiment involved a 48-day cultivation of soybean (*Glycine max* (L.) Merr.) in the soil amended with CeO_2_ NPs at 100, 500, and 1000 mg/kg, respectively. The accumulation of micro- and macro elements in the roots, root nodules, stems, pods, and leaves was assessed by chemical analysis of the plant material by ICP-OES. The results of the experiment clearly indicated that CeO_2_ NPs did not affect the level of Zn and Fe accumulation in individual parts of soybean. However, nanoceria reduced the uptake of Mo and Cu by the root, and the levels of Mn and S in individual plant parts depended on the concentration of CeO_2_ NPs in the soil. The authors also concluded that nanometric cerium(IV) oxide can modify the nutritional value of plants. They observed the reduction of Na and Al in soybean pods. The CeO_2_ NPs treatments disturbed the accumulation of Ca, Mg, P, K, and S. Other crop species have also shown evidence of the effect of nanometric cerium (IV) oxide on mineral absorption by plants. The inductively coupled plasma optical emission spectrometry (ICP-OES) method was used by Rico et al. [27] to examine the impact of CeO_2_ NPs on the concentrations of specific micro- and macronutrients in the leaves and seeds of barley (*Hordeum vulgare* L.) grown for 169 days in contact with nanoparticles. Chemical analysis revealed that CeO_2_ NPs did not impact the levels of Al, B, Ca, Mg, and Mn in the leaves or B and Na in the seeds at the investigated concentration range (125–500 mg/kg). Compared to control plants, the accumulation of K, Cu, and Zn in the leaves was higher in proportion to the increasing CeO_2_ NPs concentration in the soil. Growing plants in soil enriched with nanoparticles not only altered the standard pathways of element uptake and accumulation by the plants but also modified the complex processes controlling the content of individual mineral nutrients in barley grains. It can be proved by the much higher accumulation of Ca, Fe, P, and S at the relatively low concentration of CeO_2_ NPs (125 mg/kg), as well as Cu, K, Mg, Mn, and Zn at the medium dose (250 mg/kg). At the highest stress agent dose (500 mg/kg), it was observed that the plants did not produce fully mature spikes and, consequently, grain. According to the authors, this may be related to a noticeable increase in nutrient uptake, which negatively affected spike and grain formation processes. The effect of nanometric CeO_2_ on micro- and macronutrient accumulation levels was also investigated in maize (*Zea mays* L.) grown in contact with the nanoceria to full plant maturity (84 days) [38]. Chemical analysis showed that CeO_2_ NPs in the examined concentration range did not affect Fe or Ca levels in fully developed maize cobs. At the same time, the contents of Zn, B, Mn, Mg, S, P, and K were significantly lower compared to the results obtained for control plants. The results suggest that the elemental composition of maize generative organs was modified by CeO_2_ NPs. However, in the tested NPs concentrations (400 and 800 mg/kg), the Ce translocation to shoots was negligible. Zhao et al. [33], who investigated the elemental composition of cucumber (*Cucumis sativus*) fruit, found a negative effect of CeO_2_ NPs only for Mo accumulation. However, at the highest nanoparticle concentration tested (800 mg/kg), an increase in Mg levels in the edible part of the cucumber was observed, probably due to the promotion of the expression of aquaporins or other Mg^2+^ transporters by nanoceria. Rico et al. [48] demonstrated that CeO_2_ NPs significantly modify the accumulation of elements in the roots and leaves of wheat (*Triticum aestivum*), with less effect on their levels in the grain. In the nanoparticle concentrations range tested (125–500 mg/kg), the levels of K, P, Ca, Mg, Na, Zn, and Cu in grain were comparable to those determined for control plants. In contrast, CeO_2_ NPs were found to have a negative effect on the levels of S and Na in wheat grains. While the leaves of wheat treated with nanometric CeO_2_ only accumulated higher amounts of P and Mg, the roots showed much higher levels of K, P, S, Na, and Mn. The extent of these changes was closely related to the concentration of nanoparticles introduced into the soil. Ma et al. [54] reported that CeO_2_ NPs interfere with the element uptake and accumulation in thale cress (*Arabidopsis thaliana* (L.) Heynh.). The inductively coupled plasma optical emission spectrometry (ICP-OES) results showed that CeO_2_ NPs added to the nutrient solution reduced Fe and Mn uptake as well as Mn and K accumulation in plant shoots. As suggested by the authors, the low Fe accumulation may be due to a perturbation in the expression of Fe transport proteins (IRT1, IRT2) under the influence of subjected NPs. As the Fe uptake system in plants is not very selective, it is possible that Ce is taken up together with Fe on a cotransport basis. Therefore, the plant recognizing CeO_2_ NPs avoids Ce uptake by regulating IRT expression. In addition, at the highest CeO_2_ NPs concentration tested, P levels were found to be much lower in both roots and green parts compared to those detected in control plants. The roots of plants grown in contact with CeO_2_ NPs also had higher Ca levels than the reference. Ca^2+^ ions act as secondary messengers, activating plant defense mechanisms in response to stress factors. As the authors explain, CeO_2_ NPs-induced reactive oxygen species are able to stimulate Ca^2+^ ion channels, consequently increasing the uptake and accumulation of this macronutrient by the root. In the tested NPs concentrations (250 and 1000 mg/L), no interference in the uptake of Mg, N, S, Zn, or B was found [54].

### 2.3. Photosynthesis

Photosynthesis is a key process that determines the yield of plants and is, at the same time, very sensitive to environmental stress factors [66]. For this reason, the photosynthetic efficiency of plants is the main indicator of their ability to adapt to stress conditions.

A reduction in chlorophyll content was observed in radish (*Raphanus raphanistrum* subsp. *sativus* (L.) Domin)) grown in Hoagland’s medium supplemented with nanometric CeO_2_ at 10 mg/L [51]. The relative chlorophyll level in the green parts of these plants was about 16% lower as compared to the control. However, CeO_2_ NPs did not affect the quantum or mechanical efficiency of photosystem II. The reduction in chlorophyll content was explained by a deficiency of Mg and Fe, key elements for the synthesis of green pigment, induced by impaired uptake of nutrients by the roots. Priester et al. [45] noticed a decrease in the chlorophyll level without effects on photosynthetic parameters (F_v_/F_m_, ΦPSII) in soybean (*Glycine max* (L.) Merr.) grown in soil supplemented with CeO_2_ NPs at concentrations of 100, 500, and 1000 mg/kg, respectively. The observed decrease in chlorophyll *a* and chlorophyll *b* levels was not proportional to Ce concentration in the growth medium. The lowest green pigment content was found in the leaves of plants grown in soil with a relatively low nanoceria concentration (100 mg/kg). The detailed studies of wheat (*Triticum aestivum* L.) grown by the conventional soil method under field conditions [50] clearly show the impairment of chlorophyll synthesis induced by nanometric CeO_2._ Plants treated with nanoparticles at a concentration of 400 mg/kg showed chlorophyll *a* and chlorophyll *b* content 32% and 29% lower, respectively, than control. The decrease in the green pigment level was correlated with changes in chloroplast structure and thylakoid disorganization in mesophyll cells of leaves, confirmed by transmission electron microscopy (TEM). As suggested by the authors, these changes are an important mechanism by which plant cells adapt to the regulation of light absorption, and which prevents excessive damage under stress conditions [50]. A reduction in the content of photosynthetic pigments was also observed in the leaves of alfalfa (*Medicago sativa* L.) grown in soil supplemented with CeO_2_ NPs at concentrations of 250, 500, and 750 mg/kg [25]. The observed inhibition of chlorophyll *a* and chlorophyll *b* synthesis was not dependent on the dose of the nanoparticles in the growth medium. Changes in leaf carotenoid levels were only observed at a concentration of 250 mg/kg. As suggested by the authors, the decrease in photosynthetic pigment levels may be due to inhibition of the activity of enzymes involved in green pigment biosynthesis. On the other hand, in thale cress (*Arabidopsis thaliana* (L.) Heynh.) suppression of chlorophyll synthesis in leaves was only found at nanoceria concentrations of 1000 and 3000 mg/L, whereas relatively low concentrations of CeO_2_ NPs (<500 mg/L) did not affect the photosynthetic pigment content in leaves [55]. In addition, transmission electron microscopy images indicated a remarkable reduction in chloroplast size under the influence of CeO_2_ NPs (1000 and 3000 mg/L) and proved the presence of NPs clusters in the cytoplasm of leaf cells [55]. The low content of pigments in wheat leaves was also demonstrated by Abbas et al. [49], based on a hydroponic cultivation of wheat in 25% Hoagland solutions. A 20-day exposure of wheat roots to CeO_2_ NPs reduced the synthesis of chlorophyll *a* and chlorophyll *b*, proportional to the increase in NPs supplementations (100, 500, 1000, and 2000 mg/L). Relatively low doses of NPs (100, 500 and 1000 mg/L) showed a positive effect on the net photosynthesis rate, correlated with an increase in stomatal conductance (g_s_) in the leaves of these plants. The toxic effect of the nanoparticles was observed at a concentration of 2000 mg/L. The promotion of photosynthesis and other physiological processes in wheat was explained by the possible activation of heat shock proteins (HSPs) by CeO_2_ NPs, as well as their specific properties mimicking the action of antioxidative enzymes. In turn, high doses of the nanoparticles are responsible for the increased synthesis of ROS, thereby causing damage to the stomata structure. A positive effect of relatively low concentrations of CeO_2_ NPs on photosynthesis was also found in soybeans (*Glycine Max* L. Merr.) grown by the conventional soil method [43]. A nanoceria concentration of 100 mg/kg was found to be optimal in terms of promoting photosynthesis. A three-week exposure of the plants to nanoceria resulted in a considerable increase in g_s_, net photosynthesis rate (P_Nmax_), water use efficiency (WUE), maximum carboxylation rate (V_cmax_), and maximum rate of photosynthetic electron transport (*J*_max_) compared to the values obtained for the control plants. In addition, a much higher stomatal conductance was found in plants treated with polyvinylpyrrolidone (PVP)-coated CeO_2_ nanoparticles (ζ potential −51.57 mV) compared to those with an uncoated surface (ζ potential +45.13 mV). These results indicate that CeO_2_ NPs can promote gas exchange and photosynthetic efficiency by enhancing photosynthetic light reactions, NADPH synthesis, and RuBP regeneration, as well as activating the Rubisco enzyme, which catalyzes CO_2_ fixation in the light-independent phase. At a CeO_2_ NPs concentration of 500 mg/kg, limitations in photosynthesis were observed, manifested by a decrease in the parameters g_s_, WUE, quantum yield (*ϕ*), V_cmax_, J_max_, and mesophyll conductance (g_m_), below the values obtained for untreated plants. Compared to control, nanoceria caused an increase in chlorophyll *a* content in leaves, with a simultaneous reduction in chlorophyll *b* biosynthesis. This effect was found at 100 and 500 mg/kg CeO_2_ NPs concentrations. Inhibition of chlorophyll *a* production is a common response of plants grown under stress conditions. As a result, plants produce more chlorophyll *b* to compensate for the amount of light absorbed [43]. The beneficial role of CeO_2_ NPs on photosynthesis was also detected in the pea (*Lathyrus oleraceus* Lam.), as demonstrated by Skiba et al. [2,40]. The hydroponic cultivation of plants with nanoceria supplementations (100 mg/L) increased leaf net photosynthesis (40%), stomatal conductance (36%), and water use efficiency (30%) as related to the control [2]. Nanoceria also stimulated biochemical parameters of photosynthesis in pea, V_cmax,_ and *J*_max_. Presumably, this effect was initiated by the catalytic properties of CeO_2_ NPs, which accelerate the photochemical phase of photosynthesis. Moreover, the authors proved that nanoceria moderate ZnO NPs toxicity by protecting the photosynthetic apparatus in pea leaves from oxidative stress triggered by excess Zn [40].

No significant changes in the chlorophyll content were found in radish ((*Raphanus raphanistrum* subsp. *sativus* (L.) Domin) cultivated in soil supplemented with CeO_2_ NPs (62.5, 125, 250 and 500 mg/kg) [52]. No statistically significant changes were noted under nanoceria treatments in terms of net photosynthesis (A), transpiration (E), or stomatal conductance (g_s_). Vittori Antisari et al. [28] demonstrated that CeO_2_ nanoparticles in a concentration corresponding to 80 mg (Ce)/kg did not affect the gas exchange parameters and level of photosynthetic pigments in the leaves of basil (*Ocimum basilicum* L.). The lack of effect of nanoceria on the content of photosynthetic pigments in soybean (*Glycine Max* L. Merr.) was also reported by Cao et al. [42]. The plants were grown in soil with the addition of two types of CeO_2_ nanoparticles (PVP-coated and -uncoated) at 100 mg/kg concentration. Despite the level of total chlorophyll in leaves comparable to the control series, the intensity of photosynthesis was significantly higher than that of plants cultivated without the stress factors. This effect was associated with increased leaf stomatal conductance (g_s_). CeO_2_ NPs also altered the biochemical parameters of photosynthesis by increasing the maximum rate of photosynthetic electron transport (*J*_max_), maximum carboxylation rate (V_cmax_), and mesophyll conductance (g_m_). Interestingly, the promotion of photosynthesis by nanometric CeO_2_ was pronounced in plants grown in soil with sufficient moisture (>85%). The physiological response of plants to water stress is stomatal closure, which prevents transpirational water loss, which also results in reduced CO_2_ uptake. Thus, the limited availability of water may reduce the promotion of photosynthesis by CeO_2_ NPs. The influence of the surface modification of the nanoparticles, and their surface charge, did not differentiate the effect caused by CeO_2_ NPs on photosynthesis parameters related to energy absorption and transport. However, according to the authors, modification of the nanoparticle surface may play an important role in the mechanism of CO_2_ fixation by plants [42].

Rossi et al. [30] found an increase in the content of chlorophyll in the leaves of canola (*Brassica napus* L.) as a result of treating plants with nanometric CeO_2_. However, the higher level of photosynthetic pigments in the leaves compared to the control plants was not correlated with an increase in photosynthesis efficiency. After 40 days of canola cultivation at 200 mg/kg CeO_2_ NPs, a significant decrease in net photosynthesis rate was noted. A higher nanoceria dose (1000 mg/kg) significantly influenced the process of stomatal aperture, increasing the stomatal conductance. The photosynthetic light response curve registered for nanoceria concentration (1000 mg/kg) proved the higher efficiency in the use of light energy by photosystem II compared to the control. It was also reported by Rossi et al. [29] that a nanoceria concentration of 500 mg/kg supported PSII processes (an increase in the F_v_/F_m_ parameter). A similar effect was observed in soybean (*Glycine Max* (L.) Merr.) grown in sand with CeO_2_ NPs (500 mg/kg) saturated with 25% Hoagland solutions [41]. Chlorophyll fluorescence measurements proved the positive effect of nanoparticles on the photochemical efficiency of PSII, resulting from an increase in the value of the F_v_/F_m_ parameter. In addition, much higher levels of chlorophyll *b* were found in the leaves of plants treated with CeO_2_ NPs compared to the control series, while the amount of chlorophyll *a* did not change. The promotion of green pigment biosynthesis by CeO_2_ NPs was also reported in barley (*Hordeum vulgare* L.) grown in the conventional soil method [27]. The total time of cultivation was 169 days, while this effect was observed after 48 days of plant contact with the stressor and was independent of its concentration in the soil medium (125, 250 and 500 mg/kg).

Nanometric CeO_2_ did not affect the gas exchange parameters (A, E, g_s_) of maize (*Zea mays* L.), either at its initial growth phase [37] or in the mature stage [38]. There were also no significant changes in chlorophyll content between the different variants of the experiment. These results were confirmed by two independently carried out experiments. A similar effect was observed in cucumber (*Cucumis sativus*) grown in soil for 53 days at the nanoceria concentrations (400 and 800 mg/kg) [32]. Marchiol et al. [26], who monitored gas exchange in barley (*Hordeum vulgare* L.), demonstrated that CeO_2_ NPs are able to modify photosynthesis only during the initial phase of plant growth. At this stage, CeO_2_ NPs at 500 mg/kg enhanced the photosynthesis rate with a simultaneous increase in stomatal conductance and transpiration rate. In addition, ultrastructural analyses on leaf tissues by TEM led to the detection of CeO_2_ NPs in the leaf parenchyma, in the stroma of chloroplast, and the vacuoles.

Majumdar et al. [35] examined the impact of soil organic matter on the photosynthesis process in kidney beans (*Phaseolus vulgaris* L.) exposed to CeO_2_ NPs. Their experiment was carried out on plants grown in low organic matter (LOMS) and high organic matter (OMES) soils. It was shown that the amount of organic matter in the soil and the concentration of nanoparticles had a significant effect on the level of photosynthetic pigments in bean leaves. In plants grown in LOMS at a CeO_2_ NPs concentration of 250 mg/kg, the level of chlorophyll *a* was much lower than in the control plants. The other treatments did not alter the levels of chlorophyll *a*, chlorophyll *b,* or carotenoids. Remarkable limitations in the biosynthesis of photosynthetic pigments were observed in plants cultivated in OMES. Monitoring of gas exchange parameters revealed a significant effect of soil type and CeO_2_ concentration with respect to transpiration rate (E) and stomatal conductance (g_s_). Only in plants grown in OMES was an increase in E and g_s_ observed, without notable changes in the value of leaf net photosynthesis rate (P_n_). According to the authors, organic matter facilitated the translocation of Ce to leaves, which may account for the much greater impact of CeO_2_ NPs on the gas exchange process found in plants grown in OMES.

### 2.4. Oxidative Stress

The impact of particular stressors on plant cells, regardless of their nature, leads to uncontrolled overproduction of reactive oxygen species, which results in secondary stress, called oxidative stress. From the chemical point of view, ROS are products of successive stages of molecular oxygen reduction, generated by aerobic metabolic processes. These compounds include the superoxide anion radical (O_2_^•−^), the hydroxyl radical (•OH), as well as hydrogen peroxide (H_2_O_2_) and singlet oxygen (^1^O_2_) [67]. Considering the cellular locations of ROS generation, particularly favored sites are the thylakoid membranes in chloroplasts, the mitochondrial membrane, and peroxisomes [68]. In biological systems, ROS also plays a key role in cellular signal transduction, which enables plant responses to environmental stimuli [69,70]. Whether the function of these highly reactive molecules is signaling or destructive towards biomolecules depends on the balance between their production and scavenging or detoxification [71]. The level of ROS in plant cells can be controlled by both enzymatic and non-enzymatic antioxidant systems [72,73,74]. The latter is considered a secondary line of plant defense. In the literature, the effect of nanoparticles on oxidative stress in plants is mostly determined based on the activity of antioxidant enzymes such as superoxide dismutase (SOD), which converts O_2_^•−^ to H_2_O_2_, peroxidase (POD), catalase (CAT), guaiacol peroxidase (GPOX), and ascorbate peroxidase (APOX), which plays a crucial role in the conversion of H_2_O_2_ into H_2_O [75]. In addition to enzymes, low molecular weight metabolites including ascorbic acid, glutathione (GSH), and tocopherol form an important part of abiotic stress response in plants. Another marker of oxidative stress is malondialdehyde (MDA), a product of lipid peroxidation. Additionally, the content of certain secondary metabolites, such as simple phenolic compounds and flavonoids, is very often determined, as phenolic functional groups act as free radical scavengers. In some studies, the antioxidant capacity of plants treated with nanoparticles has also been evaluated by investigating their effectiveness in deactivating free radicals. Analytical techniques used to assess antioxidant properties include chemical methods based on reactions between the antioxidant and model free radicals (DPPH, ABTS) or metal ions (e.g., FRAP assays) [76]. A number of studies demonstrate that CeO_2_ nanoparticles are prone to induce the release of ROS and interfere with plant antioxidant defense mechanisms. Additionally, nanoceria presents enzyme-like activity and is considered a nanoenzyme for plant abiotic stress tolerance due to its enzyme-like mimetic activity, including antioxidant and oxidant actions in plants [77].

The ability of CeO_2_ NPs to generate ROS in plant tissues was confirmed in soybean (*Glycine max* (L.) Merr.) by Priester et al. [45]. The total content of ROS in leaves was determined by a fluorometric method involving the staining of plant tissues with DCFH-DA (2′,7′-dichlorofluorescin diacetate), which is oxidized to DCF (2′,7′-dichlorofluorescein) with fluorescent properties in the ROS environment. The measurements performed after 47 days of plant exposure to nanometric CeO_2_ showed that the increase in ROS concentration in leaves was in direct proportion to the increase in the amount of nanoparticles applied to the soil. However, lipid peroxidation (determined as MDA levels in plant tissues) was not dependent on the Ce dose in the soil and was ~50% higher than in control plants. Overproduction of H_2_O_2_ in plant tissues exposed to nanometric CeO_2_ was also found in thale cress (*Arabidopsis thaliana* (L.) Heynh.) as studied by Yang et al. [55]. In the range of tested CeO_2_ NPs concentrations (100, 200, 500, 1000 and 2000 mg/L), much higher H_2_O_2_ levels were found in roots compared to the control series, while in shoots this effect was evident at relatively high stressor doses (>1000 mg/L). Similar associations have been reported by authors for MDA levels in plant tissues. In contrast, the results of an experiment conducted by Rico et al. [27] proved that CeO_2_ NPs at 500 mg/kg increased the H_2_O_2_ level in barley (*Hordeum vulgare* L.), while relatively low concentrations of nanoparticles (125 mg/kg) reduced the formation of ROS. The low contents of H_2_O_2_ in plant leaves were associated with increased activity of ascorbate peroxidase (APOX) and dehydroascorbate reductase (DHAR). The overproduction of ROS found at 500 mg/kg CeO_2_ NPs suggests an increase in oxidative stress. H_2_O_2_ content was monitored in shoots of maize (*Zea mays* L.) exposed to nanometric CeO_2_ by Zhao et al. [37]. The obtained results showed that increased H_2_O_2_ production was found only in the initial phase of plant growth (10 days after the treatments with NPs), while subsequent measurements showed a gradual decrease in ROS, which eventually reached the level observed for untreated plants. The same pattern was observed for measurements of antioxidant enzyme activity, catalase (CAT), and ascorbate peroxidase (APOX). These results imply that adaptation processes in plants are gradually initiated to protect the cells from the damaging effects of oxidative stress. The initial increase in H_2_O_2_ contents and APOX activity in tissues were proportional to the final concentrations of CeO_2_ NPs in the soil. Considering the content of Ce in maize shoots, which was negligible during the initial phase of plant growth, the authors conclude that the symptoms of oxidative stress at this time did not result directly from the accumulation of CeO_2_ NPs in green parts, but from NPs contact with the roots. However, no lipid peroxidation or changes in cell membrane integrity were observed in the concentration range studied, which suggests that antioxidant enzymes were effective in eliminating excess of ROS. The authors also presented results on the levels of HSP70 proteins, which are crucial for maintaining cellular homeostasis, especially under stress conditions. Contact of roots with CeO_2_ NPs increased HSP70 levels in the underground part of the plants, and this effect persisted throughout the treatment period. Ma et al. [54] confirmed the overproduction of ROS in thale cress (*Arabidopsis thaliana* (L.) Heynh.) tissues as a response to exposure of plants to nanoceria. The authors showed increased levels of ROS in shoots at both low (250 mg/L) and high (1000 mg/L) concentrations of CeO_2_ NPs compared to the control series. In addition, increased production of H_2_O_2_ was determined by histochemical staining, while O_2_^•−^ was only detected in negligible amounts at the highest nanoparticle concentration tested (1000 mg/L). Increased activity of antioxidant enzymes such as APOX, POD, SOD, and CAT was also found in plant leaves. Moreover, non-enzymatic pathways of antioxidant protection were activated in response to plant growth in contact with CeO_2_ NPs. This was demonstrated by increased levels of phenylalanine ammonia lyase (PAL), a key enzyme in the flavonoid biosynthesis pathway, and of polyphenol oxidase (PPO), which catalyzes the biochemical conversion phase of phenolic compounds to quinones. In addition, the activities of glutathione S-transferase (GST), glutathione reductase (GR), phenylalanine ammonia lyase (PAL), and polyphenol oxidase (PPO) were highly induced in thale cress (*Arabidopsis thaliana* (L.) Heynh.) tissues exposed to 1000 mg/L CeO_2_ [54]. Changes in the levels of phenolic compounds and flavonoids under the influence of CeO_2_ NPs were also found in alfalfa (*Medicago sativa* L.) [25]. The comparison with the control series of the experiment implied that CeO_2_ NPs initiated an increase in the levels of the tested metabolites, which was dependent on the nanoparticle dose applied. These results suggest an effective activation of antioxidant defense mechanisms by plants exposed to CeO_2_ NPs.

Majumdar et al. [34] investigated indicators of oxidative stress in kidney beans (*Phaseolus vulgaris* L.) induced by supplementation of Hoagland medium with nanometric CeO_2_. According to their study, the contact with the nanoparticles did not cause significant changes in MDA content in plant organs, compared to the control. The results suggest that CeO_2_ NPs did not cause lipid peroxidation and thus did not induce oxidative damage in plant tissues. The activity of oxidative enzymes was generally unchanged after 7 days of plant cultivation in contact with nanoceria. However, long-term (15-day) contact with CeO_2_ NPs at 500 mg/L resulted in a reduction of APOX, CAT, and GPOX activities in bean roots. In the CeO_2_ NPs concentration range of 62.5–500 mg/L an increase in GPOX activity was also found in leaves. According to the authors, the decreased activity of antioxidant enzymes in roots may be caused by competitive interactions between CeO_2_ NPs and Fe, which in turn decreases the biological activity of hemoproteins (CAT, APOX, and GPOX). Another experiment conducted on *Phaseolus vulgaris* [35] demonstrated differences in the response of plants to CeO_2_ NPs depending on the organic matter abundance in the soil. The levels of CAT and APOX activities in roots, stems and leaves indicated that the plant response to stress induced by CeO_2_ was more pronounced in plants grown in organic soil. The increase in CAT and APOX activity was particularly noticeable in leaves and was closely dependent on the concentration of CeO_2_ NPs in the soil [35].

The effect of nanometric CeO_2_ on oxidative stress in plants was also studied by Rico et al. [53]. The researchers performed an experiment involving a 10-day treatment of rice (*Oryza sativa* L.) seedlings with aqueous solutions of CeO_2_ NPs at four concentration levels (62.5, 125, 250 and 500 mg/L). Compared to the control series of the experiment, CeO_2_ NPs at 62.5 mg/L caused a significant reduction in H_2_O_2_ content in rice roots. Conversely, an increase in H_2_O_2_ level was observed at the highest nanoparticle concentration (500 mg/L), while the other concentrations did not significantly affect the levels of this compound. As suggested by the authors, the reduction in hydrogen peroxide generation may result from the ability of nanoceria to ‘scavenge’ ROS, typically revealed at low concentrations of the compound in biological systems. In contrast, the increased amount of H_2_O_2_ may be due to the activity of CeO_2_ NPs, potentially acting as SOD mimetics, which can catalyze the conversion of O_2_^•−^ to H_2_O_2_. When considering the activities of individual antioxidant enzymes in rice seedlings, it can be concluded that it was strictly dependent on the concentration of the stressor. An increase in CAT and GPOX activity was observed in plants treated with CeO_2_ NPs at 62.5 mg/L, which may explain the low concentration of H_2_O_2_ in the roots of plants from this variant of the experiment. SOD activity was reduced compared to the control series at the concentration of 125 mg/L, while the dose of 250 mg/L caused the opposite effect. At the highest tested dose (500 mg/L), an increase in GPOX and APOX activity was noted, which may be the plant’s response to the intensification of oxidative stress in rice seedlings. Similar results were found in wheat (*Triticum aestivum* L.), where, at a relatively high concentration of CeO_2_ NPs (400 mg/kg) added to the soil, an increase in CAT and SOD activity was observed compared to the control series [50].

The effect of nanometric CeO_2_ on oxidative stress was also evaluated by Corral-Diaz et al. [52]. The researchers experimented on radish ((*Raphanus raphanistrum* subsp. *sativus* (L.) Domin).) grown by the traditional soil method in contact with CeO_2_ NPs at 62.5–500 mg/kg. The results showed that nanometric CeO_2_ did not induce changes in the content of flavonoids and phenolic compounds in radish tissues. However, increased antioxidant capacity, as determined by DPPH, ABTS, and FRAP assays, was observed in plants treated with nanometric CeO_2_ at 250 mg/kg. It can suggest that nanoparticles may support the plants’ ability to scavenge free radicals. Additionally, no increase in antioxidant enzyme activity was observed. In contrast, increased CAT and APOX activity was recorded in radish storage root at 125 mg/kg CeO_2_ NPs. APOX activity was also elevated at a Ce concentration of 500 mg/kg.

Gui et al. [36] observed a significant decrease in the enzymatic activity of superoxide peroxidase and dismutase in the roots of lettuce (*Lactuca sativa* L.) cultivated in contact with the highest tested CeO_2_ NPs concentration (1000 mg/kg). The lower studied concentrations (50 and 100 mg/kg) did not have a significant impact on the activity of these enzymes. Furthermore, at 1000 mg/kg, the authors determined a high level of MDA in roots, which may indicate damage to the cell membrane caused by ROS. Tassi et al. [46] proved that nanometric CeO_2_ (100, 200, 400 and 800 mg/kg) has no impact on the antioxidative enzyme activity in the leaves of sunflowers (*Helianthus annuus* L.), grown by the traditional soil method.

## 3. Conclusions

The impact of cerium oxide nanoparticles on plants has been the subject of several studies in recent years. Unfortunately, no common mechanism for the interaction of nanoparticles with plants has been developed. However, there is a general agreement that nanoceria has a specific effect on plants. It depends mainly on the dose and characteristics of the NPs. The growth medium and plant species are also primary concerns. In parallel with toxicity, usually manifested at high concentrations of CeO_2_ NPs, fertilizing effects were also reported. The latter prompts the photosynthesis process, which is crucial in the production of biomass. Cerium(IV) oxide nanoparticles considered as metabolic stimulators would play an important role, especially for crop plants. The development of formulations for commercially available nanoproducts that could be successfully applied in contemporary agriculture is still a challenge. Obviously, there is a strong need for the standardization of methodologies and cultivation conditions, which will ensure a high comparability of experimental results [78]. A variety of experimental conditions, a huge number of plant species, and a plethora of NPs are among the major challenges as far as this issue is concerned.

## Figures and Tables

**Table 1:** Summary of studies conducted with CeO_2_ NPs, including their impact on plant growth, photosynthesis, mineral nutrition, and oxidative stress.

Test Plant	Characterization of CeO_2_ NPs	Concentrations of CeO_2_ NPs	Treatment	Impact on				Ref.
				Growth Parameters	Photosynthesis	Mineral Nutrition	Oxidative Stress	
Alfalfa (*Medicago * *sativa* L.)	8 ± 1 nm, 231 ± 16 (DLS)	250, 500, 750 mg/kg	Soil, 30 days	-	▪ Decreased chlorophyll *a* and chlorophyll *b* contents ▪ Decreased carotenoid content at 250 mg/kg	-	Concentration-dependent impacts of CeO_2_ NPs on phenolic compounds and flavonoid levels in roots and shoots	[25]
Barley (*Hordeum * *vulgare* L.)	32.6 ± 20.7 nm (AFM), 174 ± 1 nm (DLS), ζ-potential 0.027 ± 0.064 mV (DLS)	500, 1000 mg/kg	Soil, 92 days	▪ Plants had a longer vegetative period ▪ Decrease in the number of tillers, leaf area, and the number of spikes per plant	▪ Enhanced leaf photosynthesis rate and transpiration at booting phenological stages (500 mg/kg)	-	-	[26]
Barley (*Hordeum * *vulgare* L.)	8 ± 1 nm (TEM), 231 ± 16 (DLS)	125, 250, 500 mg/kg	Soil, 169 days	▪ Decrease in the number of spikes but an increase in spike length and number of spikelets per spike at 250 mg/kg ▪ Increase in plant height, dry shoot biomass, and water content at 500 mg/kg ▪ Inhibition of grain production at 500 mg/kg	▪ Increase in chlorophyll content	▪ No effect on Al, B, Ca, Mg, and Mn contents in leaves ▪ No effect on B and Na contents in grains ▪ Leaves: ↑ K, Cu, Zn, Fe; ↓ P, S, Na, ▪ Grains: ↑ Ca, Fe, P, Mn	▪ Decrease in H_2_O_2_ content in leaves at 125 mg/kg ▪ Increase in H_2_O_2_ content in leaves and K leakage at 500 mg/kg ▪ No effect on CAT activity	[27]
Basil (*Ocimum * *basilicum* L.)	15–30 nm, ζ-potential 44.5 mV (DLS)	80 mg/kg	Soil mixture, 28 days	▪ No effect on vegetative growth	▪ No effects on chlorophyll content ▪ No effects on gas exchange parameters	-	-	[28]
Canola (*Brassica * *napus* L.)	20–110 nm (TEM), ζ-potential −51.8 mV (DLS), PVP-coated	500 mg/kg	Sand, 30 days	▪ No effect on leaves and roots biomass	▪ Increased maximum quantum efficiency of photosystem II (Fv/Fm)	-	-	[29]
Canola (*Brassica * *napus* L.)	20–110 nm (TEM), ζ-potential −51.8 mV (DLS), PVP-coated	200, 1000 mg/kg	Sand plus LECA (1:1, *v*/*v*), 40 days	▪ Increase in fresh and dry biomass of roots at 1000 mg/kg ▪ No effect on leaf biomass	▪ Increased chlorophyll content, decreased net photosynthesis rate at 200 mg/kg ▪ Increased gs at 1000 mg/kg ▪ Increased maximum quantum efficiency of photosystem II (Fv/Fm)	▪ Increase in Mg content in leaves	-	[30]
Cilantro (*Coriandrum sativum* L.)	8 ± 1 nm (TEM), 231 ± 16 (DLS)	62.5, 125, 250, 500 mg/kg	Soil, 30 days	▪ Promotion of root and shoot growth at 125 mg/kg ▪ No effect on dry biomass	-	-	▪ Higher activity of CAT in shoots and APOX in roots at 125 mg/kg	[31]
Cucumber (*Cucumis * *sativus* L.)	8 ± 1 nm (TEM), 231 ± 16 (DLS)	400, 800 mg/kg	Mixture of sand soil, sand, and perlite (1:1:3, *v*/*v*), 53 days	▪ No effect on biomass production, shoot length, and leaf area ▪ Decrease in fruit weight at 800 mg/kg	▪ No effects on gas exchange parameters ▪ No effect on chlorophyll content	▪ No effect on K, Ca, S, P, Na, Fe, Cu, Mn, and Zn accumulations in fruit ▪ Increased Mg allocation in fruit at 400 mg/kg	▪ Decreased phenolic compound levels at 800 mg/kg	[32,33]
Kidney bean (*Phaseolus * *vulgaris* L.)	8 ± 1 nm, 231 ± 16 (DLS)	62.5, 125, 250, 500 mg/L	Hydroponic conditions, Hoagland solution, 15 days	▪ Dose-dependent effects on biomass productions	▪ No effect on chlorophyll content	-	▪ No effect on MDA contents and lipid peroxidation ▪ Higher activity of APOX, GPOX ▪ Decrease in enzyme activity at high NPs concentrations and long exposition time	[34]
Kidney bean (*Phaseolus * *vulgaris* L.)	8 ± 1 nm, 231 ± 16 (DLS)	62.5, 125, 250, 500 mg/kg	Low organic matter soil (LOMS), organic matter enriched soil (OMES), 52 days	▪ No effect on roots and shoot length ▪ Increase of leaf area in plants growing in LOMS	▪ Chlorophyll increased at 250 mg/kg LOMS ▪ Decrease in chlorophyll a (125, 250 mg/kg, OMES) and carotenoids (62.5–250 mg/kg, OMES) ▪ Net photosynthesis increased at 62.5 mg/kg, LOMS ▪ E and gs increased, OMES	▪ No effect on Al, B, Cu, Mn, Fe, and Zn uptake by root	▪ No effect on CAT and APOX activity in roots ▪ Higher activity of APOX (125, 250 mg/kg) and CAT (62.5 mg/kg) in leaves, OMES	[35]
Lettuce (*Lactuca * *sativa* L.)	< 25 nm (TEM), 149.1 ± 23.5 nm (DLS), ζ-potential 13.8 ± 4.0 mV (DLS)	50, 100, 1000 mg/kg	Soil, 30 days	▪ Increased fresh biomass of roots and shoots at 100 mg/L ▪ Decreased dry biomass of roots at 1000 mg/kg	-	-	▪ Decreased activity of SOD and POD in roots at 1000 mg/kg ▪ Increased MDA content in roots at 1000 mg/kg	[36]
Maize (*Zea mays* L.)	10 ± 1 nm, 2124 ± 59 nm (DLS), ζ-potential −22.8 ± 4.5 mV (DLS)	400, 800 mg/kg	Soil, 20 days	-	▪ No effect on gas exchange parameters (A, E, g_s_)	-	▪ Increase in H_2_O_2_ content and activity of CAT and APOX in shoots at 10 days ▪ Increase in HSP70 level in roots ▪ No effect on lipid peroxidation and membrane integrity	[37]
Maize (*Zea mays* L.)	8 ± 1 nm, 231 ± 16 (DLS)	400, 800 mg/kg	Mixture of sand soil, sand, and perlite (1:1:3, *v/v*), 84 days	▪ No effect on biomass production, shoot length, leaf area, and yield of corn	▪ No effect on gas exchange parameters (A, E, g_s_)	▪ No effect on Fe, Ca contents in cobs, decreased contents of Zn, B, Mn, Mg, S, P, and K in fully developed cobs	-	[38]
Mesquite (*Neltuma velutina* (*Wooton*) *Britton* & *Rose*)	8 ± 1 nm, 231 ± 16 (DLS)	500, 1000, 2000, 4000 mg/L	Hydroponic conditions, modified Hoagland solution, 15 days	▪ No visible signs of toxicity, like chlorosis or growth inhibition	-	▪ No effect on uptake and accumulation of macronutrients; decrease in Cu and Mn uptake; Zn uptake increased at 500 mg/L and decreased at >1000 mg/L	▪ Higher APOX activity in roots at >2000 mg/L ▪ Higher CAT activity in leaves at 4000 mg/L	[39]
Pea (*Lathyrus * *oleraceus* Lam.)	26 ± 14 nm (TEM), ζ-potential −41.55 ± 1.13 mV (DLS)	100, 200, 500 mg/L	Hydroponic conditions, Hoagland solution, 12 days	▪ Decreased fresh biomass of roots and shoots at 500 mg/L ▪ No effect on dry biomass	▪ Chlorophyll a, chlorophyll b, and carotenoids increased at 500 mg/L ▪ Enhanced net photosynthesis (A), E, and WUE at 100 mg/L	▪ Root: ↑ Ca; ↓ Cu, Zn, Mn, Fe, and Mg accumulation ▪ Shoot: ↓ Cu, Zn, Mn, Fe, Mg, and Ca accumulation	-	[2,40]
Soybean (*Glycine max* (L.) Merr.)	41.7 ± 5.2 nm (TEM), ζ-potential −51.57 mV (DLS), PVP-coated	500 mg/kg	Sand, 30 days	▪ No effect on dry biomass of roots and shoots	▪ Increased chlorophyll b ▪ No effect on chlorophyll a ▪ Increased maximum quantum efficiency of photosystem II (F_v_/F_m_)	-	-	[41]
Soybean (*Glycine max * (L.) Merr.	PVP-coated, 6–24 nm, ζ-potential −51.57 mV (DLS), uncoated, 10–30 nm, ζ-potential 45.13 mV (DLS)	100 mg/kg	Soil under different moisture conditions, 21 days	▪ Increased fresh and dry biomass of roots and shoots	▪ No effect on chlorophyll content ▪ Increase in net photosynthesis rate and g_s_ ▪ Increase in biochemical parameters: J_max_, V_cmax,_ and g_m_	-	-	[42]
Soybean (*Glycine max * (L.) Merr.)	PVP-coated, 6–24 nm, ζ-potential −51.57 mV (DLS), uncoated, 10–30 nm, ζ-potential 45.13 mV (DLS)	10, 100, 500 mg/kg	Soil, 21 days	▪ Increased fresh biomass of roots and shoots (PVP-CeO_2_NPs) ▪ Increased fresh biomass of shoots at 500 mg/kg CeO_2_ NPs	▪ Increased chlorophyll a content, decreased chlorophyll b at >100 mg/kg ▪ Increased net photosynthesis rate and gs at 10 and 100 mg/kg ▪ Increased WUE, J_max_, and V_cmax_ at 100 mg/kg ▪ Decreased net photosynthesis rate, g_s_, WUE, J_max_, V_cmax_, g_m_ at 500 mg/kg	-	-	[43]
Soybean (*Glycine max * (L.) Merr.)	~ 8 nm	100, 500, 1000 mg/kg	Soil, 48 days	-	▪ Decreased chlorophyll a and chlorophyll b ▪ No impact on the maximum quantum efficiency of photosystem II (F_v_/F_m_)	▪ No impact on Zn, Fe accumulation; decreased uptake of Mo, Cu, Na, and Al; ▪ Mn and S levels depend on NPs concentrations	▪ Increased ROS content in leaves ▪ Increased lipid peroxidation at 100 and 500 mg/kg.	[44,45]
Sunflower (*Helianthus* *annuus* L.)	8 ± 1 nm (TEM), 1373 ± 32 nm (DLS), ζ-potential −0.62 ± 2.9 mV (DLS)	100, 200, 400, 800 mg/kg	Soil, 35 days	▪ No sign of acute toxicity ▪ No effect on biomass production	-	-	▪ No effect on SOD, CAT, and APOX activity in leaves	[46]
Tomato (*Solanum lycopersicum* L.)	20 ± 19 nm (TEM)	0.1, 1.0, 10 mg/L	Soil, 70 days	▪ No effect on germination, leaves, and flower development ▪ Positive effect on plant growth and tomato production	▪ No effect on chlorophyll content	-	-	[47]
Wheat (*Triticum * *aestivum* L.)	8 ± 1 nm, 231 ± 16 (DLS)	125, 250, 500 mg/kg	Soil, 94 days	▪ Concentration-dependent impact of CeO_2_ NPs on yield parameters ▪ Increased plant growth, shoot biomass, and grain yield at 500 mg/kg ▪ Delay in spike formation and physiological maturity in wheat	▪ No effect on chlorophyll level	▪ No effect on Ca, Zn, and Cu accumulations, ▪ Roots: ↑ K, P, S, Mn; ↓ Fe, ▪ Leaves: ↑ Mg; ↓ Fe, ▪ Grains: ↓ S, Mn.	-	[48]
Wheat (*Triticum * *aestivum* L.)	10–20 nm	100, 500, 1000, 2000 mg/L	Hydroponic conditions, 25% Hoagland solution, 20 days	▪ Increased fresh biomass of roots and shoots at 100 and 500 mg/L; reduced at 1000 and 2000 mg/L ▪ Increase in shoot growth at 500 mg/L ▪ Decreased root elongation at 100, 1000, and 2000 mg/L	▪ Decreased chlorophyll content ▪ Increased g_s_ and net photosynthesis rate at 100–1000 mg/L ▪ Decreased g_s_, E, and net photosynthesis rate at 2000 mg/L	▪ Roots: ↑ K; ↓ N, ▪ Leaves: ↑ K; ↓ N,	-	[49]
Wheat (*Triticum * *aestivum* L.)	8 ± 1 nm (TEM), 231 ± 16 (DLS)	100, 400 mg/kg	Soil, 7 months	▪ Delay in flowering period and shortened grain filling period ▪ No impact on biomass production, plant growth, and grain weight	▪ Decreased chlorophyll contents at 400 mg/kg ▪ Changes in chloroplast and thylakoid structures	-	▪ Higher activity of CAT and SOD at 400 mg/kg	[50]
Radish (*Raphanus raphanistrum* subsp. *Sativus* (L.) Domin)	10–30 nm (TEM)	10 mg/L	Hydroponic conditions, 25% Hoagland solution, 21 days	▪ No effect on dry biomass ▪ No apparent adverse effect on the growth and development of plants	▪ Decreased chlorophyll content ▪ No effect on quantum and photochemical efficiency of PSII	-	-	[51]
Radish (*Raphanus raphanistrum* subsp. *Sativus* (L.) Domin)	8 ± 1 nm (TEM), 231 ± 16 (DLS)	62.5, 125, 250, 500 mg/kg	Soil, 40 days	▪ Retarded seed germination at 500 mg/kg ▪ No effect on growth parameters ▪ No sign of acute toxicity	▪ No effect on chlorophyll content ▪ No effect on gas exchange parameters	▪ Increase in S and B uptake by roots ▪ No effect on micro- and macronutrient accumulations in leaves and storage roots	▪ No effect on phenolic compounds and flavonoid contents ▪ Higher antioxidant capacity at 250 mg/kg ▪ Increase in CAT and APOX activity at 125 mg/kg and APOX at 500 mg/kg in storage roots	[52]
Rice (*Oryza sativa* L.)	8 ± 1 nm (TEM), 231 ± 16 (DLS)	62.5, 125, 250, 500 mg/L	Petri dish, 10 days	▪ No sign of acute toxicity	-	-	▪ Decrease in H_2_O_2_ content; increase in CAT and GPOX activity at 62.5 mg/L ▪ Lipid peroxidation in roots, decrease in SOD activity at 125 mg/L ▪ Increase in SOD and GPOX activity at 250 mg/L ▪ Increase in H_2_O_2_ content; increase in APOX and GPOX activity at 500 mg/L	[53]
Thale cress (*Arabidopsis * *thaliana* (L.) Heynh.)	10–30 nm, 294.4 ± 2.5 nm (DLS), ζ-potential 43.1 ± 2.1 mV (DLS)	250, 1000 mg/L	Hydroponic conditions, 50% Murashige & Skoog solution, 25 + 5 days	-	-	▪ No effect on Mg, N, S, Zn, and B accumulations ▪ Roots: ↑ Ca; ↓ Fe, Mn, P ▪ Shoots: ↓ Mn, K, P	▪ Increased ROS content in shoots ▪ Higher activity of APOX, CAT, SOD, POD ▪ Increased GST, GR, PAL, and PPO activity at 1000 mg/L	[54]
Thale cress (*Arabidopsis * *thaliana* (L.) Heynh.)	28 nm (XRD)	100, 200, 500, 1000, 3000 mg/L	Agar plus Murashige & Skoog solution, 25 days	▪ Increase in fresh biomass of roots and shoots at <500 mg/L ▪ Sign of toxicity at >1000 mg/L	▪ No effect on total chlorophyll content at < 500 mg/L ▪ Decrease in chlorophyll at > 1000 mg/L.	-	▪ Increase in H_2_O_2_ and MDA content in roots and shoots	[55]

↑-increased in particular element accumulation; ↓-decreased in particular element accumulation.

## Supplementary Materials

The following supporting information can be downloaded at: https://www.mdpi.com/article/10.3390/ijms25074018/s1.

## Abbreviations

A	net photosynthesis
ABTS	2,2′-azino-bis(3-ethylbenzothiazoline-6-sulfonic acid) assay
AFM	atomic force microscopy
APOX	ascorbate peroxidase
CAT	catalase
DLS	dynamic light scattering
DPPH	1,1-diphenyl-2-picrylhydrazyl assay
E	transpiration
ENPs	engineered nanoparticles
FRAP	ferric reducing antioxidant power assay
F_m_	maximum fluorescence
F_v_	variable fluorescence
F_v_/F_m_	maximum quantum efficiency of PSII
g_m_	mesophyll conductance
GPOX	guaiacol peroxidase
g_s_	stomatal conductance
GSH	glutathione
HSPs	heat shock proteins
ICP-OES	inductively coupled plasma optical emission spectrometry/spectroscopy
ICP-MS	inductively coupled plasma mass spectrometry
*J*_max_	maximum rate of photosynthetic electron transport
LOMS	low organic matter soil
MDA	malondialdehyde
NADPH	reduced form of nicotinamide adenine dinucleotide phosphate
NPs	nanoparticles
OMES	high organic matter soil
P_Nmax_	net photosynthesis rate
POD	peroxidase
PSII	photosystem II
REE	rare earth elements
ROS	reactive oxygen species
Rubisco	ribulose-1,5-bisphosphate carboxylase/oxygenase
RuBP	ribulose-1,5-bisphosphate
SOD	superoxide dismutase
TEM	transmission electron microscopy
WUE	water use efficiency
XRD	X-ray diffraction
V_cmax_	maximum carboxylation rate
Φ	quantum yield
ΦPSII	PSII operating efficiency (quantum yield of PSII)
