# Supplementary material for: Root-Applied Cerium Oxide Nanoparticles and Their Specific Effects on Plants: A Review
Source: Int J Mol Sci. 2024 Apr 4;25(7):4018. doi: 10.3390/ijms25074018 (PMC11012102; doi:10.3390/ijms25074018)
Supplement: Supplementary file 1 [file ijms-25-04018-s001.zip › ijms-2898182-supplementary.pdf]

Supplementary Materials

Root-Applied Cerium Oxide Nanoparticles and Their Specific Effects on Plants: a Review

Monika Pietrzak [1,*], Elżbieta Skiba [1,*] and Wojciech M. Wolf [1]

[1] Institute of General and Ecological Chemistry, Lodz University of Technology, Zeromskiego 114, 90-543 Lodz, Poland; monika.pietrzak@p.lodz.pl (M.P.); elzbieta.skiba@p.lodz.pl (E.S.); wojciech.wolf@p.lodz.pl (W.M.W.)

* Correspondence: monika.pietrzak@p.lodz.pl ; elzbieta.skiba@p.lodz.pl ; Tel.: +48-42-631-31-23

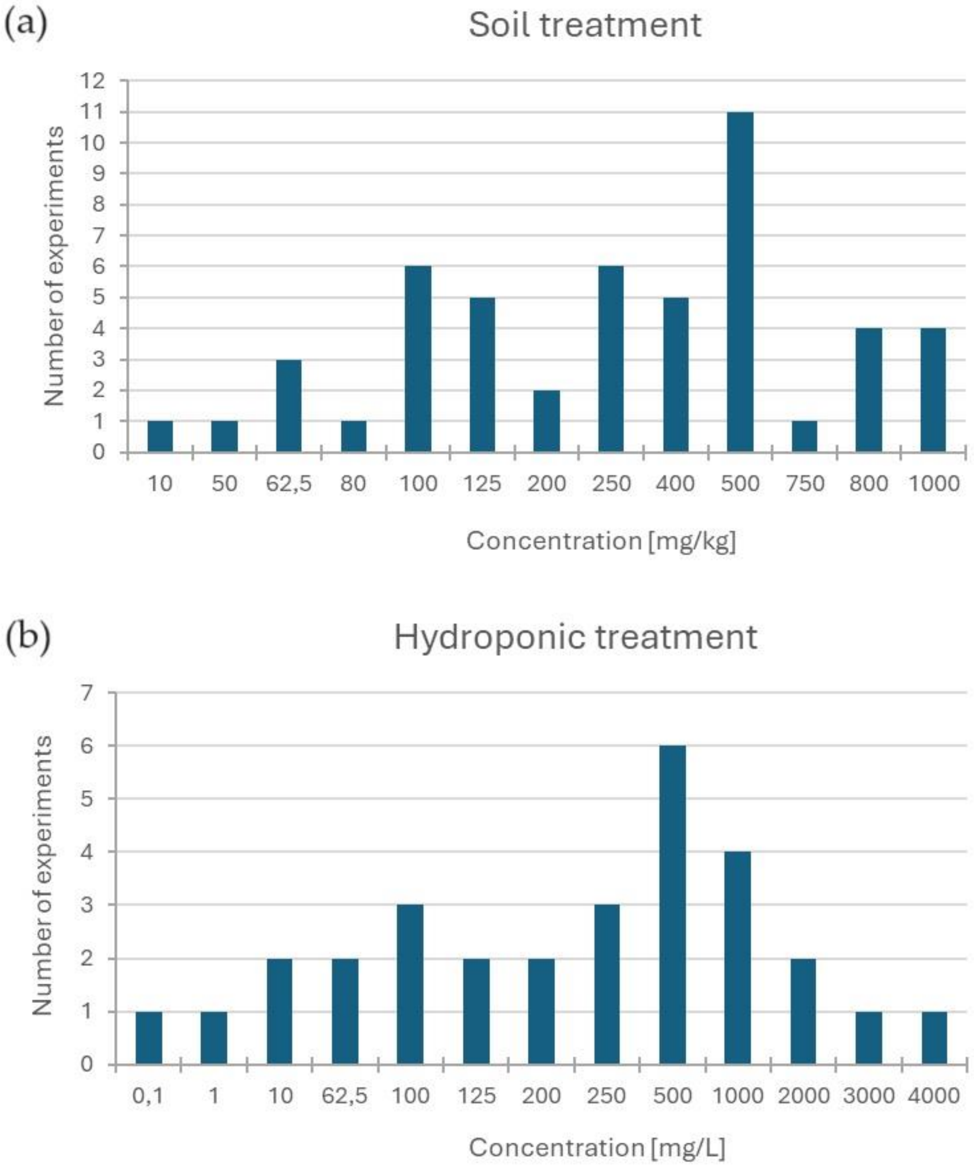

Figure S1 Distribution of nanoceria levels in soil cultivations (a) and hydroponic media (b) over papers reviewed in this work
